# Effect of Halloysite Nanotube on Mechanical Properties, Thermal Stability and Morphology of Polypropylene and Polypropylene/Short Kenaf Fibers Hybrid Biocomposites

**DOI:** 10.3390/ma13194459

**Published:** 2020-10-08

**Authors:** Piotr Franciszczak, Iman Taraghi, Sandra Paszkiewicz, Maksymilian Burzyński, Agnieszka Meljon, Elżbieta Piesowicz

**Affiliations:** Department of Materials Technologies, West Pomeranian University of Technology, Piastów av. 19, PL-70310 Szczecin, Poland; piotr.franciszczak@zut.edu.pl (P.F.); taraghi.iman@gmail.com (I.T.); maksymilian.burzynski@zut.edu.pl (M.B.); meljonagnieszka@gmail.com (A.M.); senel@zut.edu.pl (E.P.)

**Keywords:** halloysite nanotube, nano-biocomposites, mechanical properties, creep behavior, thermal stability

## Abstract

In this article, the effect of the addition of halloysite nanotube (HNT) on the mechanical and thermal stability of polypropylene (PP) and PP/kenaf fiber biocomposites has been investigated. Different volume contents of HNTs ranging from 1 to 10 vol.% were melt mixed with PP and PP/kenaf fibers. The volume content of kenaf fibers was kept constant at 30%. The morphology of HNTs within the PP matrix has been studied via scanning electron microscopy (SEM). The morphological results revealed that HNT was uniformly dispersed in the PP matrix already at a low concentration of 1 and 2 vol.%. The mechanical properties of the manufactured nanocomposites and hybrid biocomposites such as Young’s modulus, tensile strength, elongation at break, flexural modulus, flexural strength, and notched Izod strength have been measured. The results show that Young’s modulus and strengths have been improved along with the addition of low content of HNTs. Moreover, the gain of notched Izod impact strength obtained by the addition of short kenaf fibers was maintained in hybrids with low concentrations of HNTs. Finally, the thermogravimetric analysis shows that at 10% and 50% weight loss, the thermal degradation rate of the PP and PP/kenaf biocomposites decreased by the addition of HNTs.

## 1. Introduction

Halloysite nanotubes have a high amount of 1D nanotubular structures with high length-to-diameter ratio and low hydroxyl group density on the surface [1]. The HNT contains nanotubes and nanoplatelets of halloysite and similarly to montmorillonite, it has two layers of aluminosilicate. The HNTs contained in this nanoclay offer numerous benefits due to their high mechanical strength, thermal stability, and biocompatibility [2]. HNTs have been combined with polymers to obtain efficient nanocomposites (NCs) with superior advantages such as reinforcing effects, enhanced flame retardancy, and reduced thermal expansion [3,4]. There have been many reports on the influence of HNTs on the physical performance of the polymer matrix. NCs based on polypropylene (PP) and HNTs have been manufactured via melt blending processing [5]. In this case, the thermal stability of the NCs has been improved along with an increase in the content of HNTs. Lecouvet et al. [6] have proposed a water-assisted extrusion as a novel processing route to fabricate PP/HNTs NCs. The results showed that the NCs prepared via this novel method are promising candidates for flame retardant applications. Besides, Wang and Huang [7] have prepared a PP/HNTs nanocomposite through water-assisted injection molding and compression molding. The HNTs showed a direct stabilizing effect on the PP, which related to the interaction between PP macromolecules and the HNT surface.

Fiber-reinforced polymer hybrid NCs exhibit numerous exciting potential applications in economical and ecological technologies [8,9,10]. Both thermoplastic and thermoset have been combined with natural fibers and nanofillers to obtain polymer hybrid NCs [11]. Saba et al. [12,13] have studied the effect of nanofillers on the physical, structural, and thermomechanical properties of kenaf/epoxy composites produced via the hand lay-up method. Moreover, using the hot compression method, polymer hybrids with natural fibers and nanoclay have been prepared from PP, kenaf/coir fiber, and montmorillonite [14]. The interfacial interactions and adhesion of fiber and polymer matrix have been improved in the presence of nanofillers in polymer hybrid NCs. Piekarska et al. [15] have prepared a hybrid nanocomposite based on polylactide (PLA) reinforced with organic nanofillers (montmorillonite and calcium carbonate nanoparticles) and cellulose fibers. The research depicted that all nanofillers caused an increase in the storage modulus values below the glass transition temperature. Roy et al. [16] have presented a synergistic effect of short jute fiber and nanoclay on the mechanical and thermal properties of natural rubber composites. The dynamic mechanical properties and thermal stability of natural rubber/nanoclay/jute fiber hybrid composites improved synergistically. Besides, carbon nanotubes have been added to polymer composites based on natural fibers to improve the mechanical, thermal, and visco-plastic properties of the composites [17,18,19,20,21,22,23]. Similar synergistic effects in terms of mechanical performance in short fiber composites with PP matrix were investigated by Franciszczak et al., however, without the use of nanofiller [24]. Kord et al. [25] have studied the effect of multiwall carbon nanotubes (MWCNTs) on the dynamic mechanical properties of PP/reed flour composites. The obtained results revealed that the storage modulus increased with the addition of MWCNTs, while the damping properties decreased with the incorporation of MWCNTs. Moreover, the mechanical properties of PP/kenaf fiber showed an increment in the presence of MWCNTs [26].

The main objective of this study is to present the effect of HNTs on the mechanical, morphological, and thermal properties of PP/kenaf fiber. To the authors’ best knowledge, there are no reports studying the influence of HNTs on the mechanical performance of a PP/kenaf fiber composite. The proposed hybrid nano-biocomposites have been used in applications where extreme mechanical properties, high flame retardancy, and reduced thermal expansion are required. These samples can apply in household products, the automotive industry, and marine and aircraft interior parts.

## 2. Materials and Methods

### 2.1. Materials

**Figure 1:**
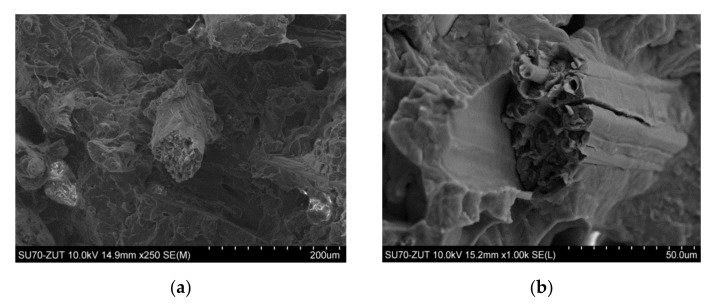
Morphology of PP/kenaf (70/30 vol.%) biocomposites at different magnifications (**a**) ×250, (**b**) ×1 k.

**Figure 2:**
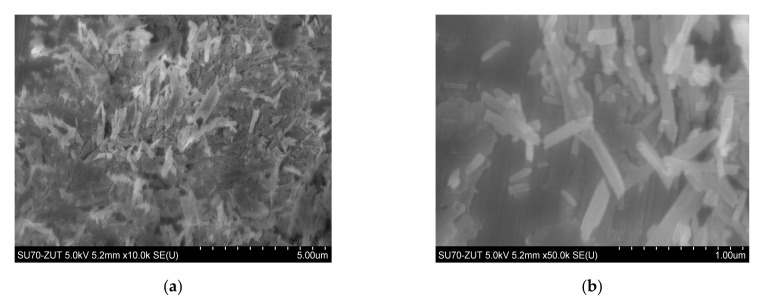
Morphology of halloysite nanotube with visible HNTs at different magnification (**a**) ×10 k, (**b**) ×50 k.

Polypropylene produced by Basell-Orlen, type HP400R (injection molding grade homopolymer), was used as the matrix. Its typical customer applications are furniture and housewares. This homopolymer exhibits good stiffness and high fluidity of Melt Flow Rate (MFR) = 25 and Melt Volume Rate (MVR) = 34 (230 °C/2.16 kg). Kenaf was planted and its fibers were prepared by water retting processes at a pilot plant in the farm of Khartoum University (Khartoum, Sudan). The processing relied on manual decortication of the bast of the outer bark of the harvested stems, which were subjected to water treatment. Afterward, they were dried at room temperature. Obtained single fibers were 1.2–1.5 m long of a bright hue containing little amounts of darker bark residues. Kenaf fibers were mechanically cut into 2 mm lengths at EKOTEX, Namysłów, Poland. Figure 1 shows the morphology of PP/kenaf biocomposites at different magnifications. HNT (under the trademark “Dunino”) in the form of a powder containing rod-shaped HNTs of diameter 100–140 nm and length 1–5 μm, bulk density 450–600 g/dm^3^ was obtained from Intermark (Gliwice, Poland). Figure 2a,b shows the scanning electron microscopy (SEM) images of the HNT at various magnifications.

### 2.2. Sample Preparation

To prepare the PP/HNTs NCs and PP/kenaf/HNTs hybrid nano-biocomposite, first, the PP/HNTs (60/40 vol.%) masterbatch with 3 vol.% of maleic anhydride-grafted PP (MAH-g-PP) in the PP matrix was manufactured to provide a good distribution of HNTs as well as enable easy feeding by the compounding. The proportional content of MAH-g-PP concerning reinforcement was kept for all manufactured composites. From the previous study [27], the optimum results for the mechanical properties of composites reinforced with cellulose-based fibers were obtained with fiber contents of ~30% of volume. The PP matrix composites for injection molding in the automotive industry are reinforced with 20–40 wt.% of natural fibers (~15–30 vol.%). PP/kenaf (30 vol.%) can be easily injection molded and higher volumetric contents of fibers reduce the flow of molten compound, thus hindering the injection process. Therefore, in this study, the PP/kenaf (30 vol.%) is used as a reference composite, and then, the effects of the addition of different weight percentages of HNTs (1, 2, 5, and 10 vol.%) in this compound are studied. Kenaf fibers were dried in a drying oven at 103 °C for about 16 h prior to the compounding. For melt mixing of PP, kenaf, and HNTs, a twin-screw extruder (counter-rotating, L/D = 23, D = 34 mm, tight intermeshing twin-screw extruder, Labor extruder LSM30 Leistritz, Nuremberg, Germany) was used. Kenaf fibers and PP (mixed with HNT masterbatch and MAH-g-PP) granulates were simultaneously fed into the extruder from two gravimetric feeders. The compositions of the PP/HNT nanocomposites (NCs) and PP/kenaf/HNT nano-biocomposites, as well as the reference PP/kenaf biocomposite, are listed in Table 1. The temperature was set from 120 to 200 °C with a 10 °C increment on each heating zone, and the screw speed was 50 RPM. The extruded strand of each polymer compound was cooled in a water bath and subsequently was pelletized. Then, the granules were dried for 16 h at 103 °C before processing by injection molding. The temperature was set from 140 to 200 °C with a 10 °C increment on each heating zone. The melt flow rate was kept at 20 ccm/s and the mold temperature was set to 10 °C.

To set the weight contents of constituents used by composite manufacturing from the planned volume contents, the following calculations using Equations (1)–(4) were done.

The theoretical density of the composite was calculated according to:

(1) $$\rho_{\mathrm{Tcomp}.}=\varphi_{m}\times \rho_{m}+\varphi_{f}\times \rho_{f}+\varphi_{n}\times \rho_{n}$$

The weight content of the PP matrix was calculated according to:

(2) $$m_{\mathrm{wt}\text{-}\%}=\frac{\varphi_{m}\times \rho_{m}}{\rho_{\mathrm{Tcomp}.}}$$

The weight content of the kenaf fibers was calculated according to:

(3) $$f_{\mathrm{wt}\text{-}\%}=\frac{\varphi_{f}\times \rho_{f}}{\rho_{\mathrm{Tcomp}}}$$

The weight content of the cellulose microfiller was calculated according to:

(4) $$n_{\mathrm{wt}\text{-}\%}=\frac{\varphi_{n}\times \rho_{n}}{\rho_{\mathrm{Tcomp}}}$$

where $\varphi_{m}$ is volume content of matrix, $\varphi_{f}$ is volume content of kenaf fibers, $\varphi_{n}$ is volume content of HNT nanoclay, $\rho_{m}$ is the density of PP matrix, $\rho_{R\;\mathrm{fib}.}$ is the density of kenaf fibers, and $\rho_{n}$ is the density of HNT nanoclay. It is worth mentioning that MAH-g-PP wax compatibilizer has the same density as the PP matrix, which simplifies the calculations.

### 2.3. Characterization

#### 2.3.1. Mechanical Properties

The tensile properties of the samples were measured using an Autograph AGS-X plus (Shimadzu, kyoto, Japan) tensile testing machine equipped with a 10 kN Shimadzu load cell, a contact extensometer, and the TRAPEZIUM X computer software (Shimadzu, Kyoto, Japan), operated at a constant crosshead speed of 1 mm/min. Measurements were performed at room temperature with a gauge length of 50 mm. According to EN ISO 527 standard, the tensile modulus, tensile strength, and elongation at break of the NCs were determined. Ten individual injection molded samples were measured for each type of manufactured material, and the results were averaged to obtain the arithmetic mean value.

#### 2.3.2. Thermogravimetric Analysis

Thermo-oxidative stability of the specimens was carried out using thermogravimetric analysis (TGA 92-16.18 Setaram, Caluire-et-Cuire, France). Measurements were performed in an oxidizing atmosphere, i.e., dry, synthetic air (N_2_:O_2_ = 80:20 vol.%). The study was conducted in the temperature range of 20 to 700 °C at the heating rate of 10 °C/min. Measurements were performed following the PN-EN ISO 11358:2004 standard.

#### 2.3.3. Izod Impact Strength

The notched impact strength was evaluated using the Izod method according to EN ISO 180/A on a Type B5102 apparatus manufactured by Zwick, Germany. The A-notch was prepared on specimens for the Izod test using a dedicated notching machine. All samples were tested at room temperature of 23 °C. The values present the averaged results for 10 tests carried out for each type of material.

#### 2.3.4. Creep Test

The creep tests were carried out on tensile specimens using a Shimadzu universal testing machine. The first mode of the creep test was done by straining the specimens with a velocity of 1mm/min until reaching 1% of strain. Afterward, the sample was kept strained at 1% for 10 min and tensile stress was recorded. In the second mode of the creep test, the samples were strained with a velocity of 1mm/min until reaching the set stress value and the reached stress was maintained for 10 min and after that, the achieved strain was recorded. The PP/HNTs NCs were strained until reaching 20 MPa, while PP/kenaf/HNTs hybrid biocomposites were strained until reaching 35 MPa (all hybrids and reference biocomposite) and 50 MPa (hybrids with 1 and 2 vol.% of HNT and reference biocomposite). Five samples were used for each material and mode of testing and the results were averaged.

## 3. Results and Discussion

### 3.1. Morphological Evaluations

**Figure 3:**
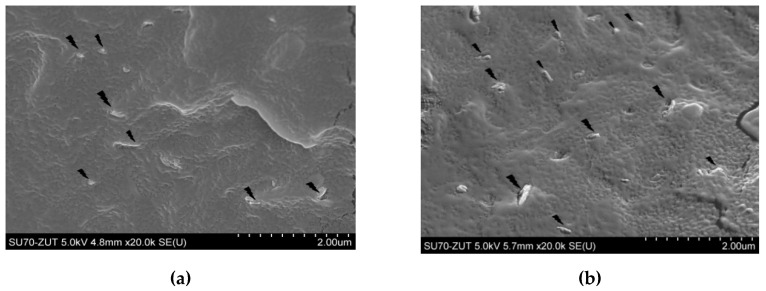

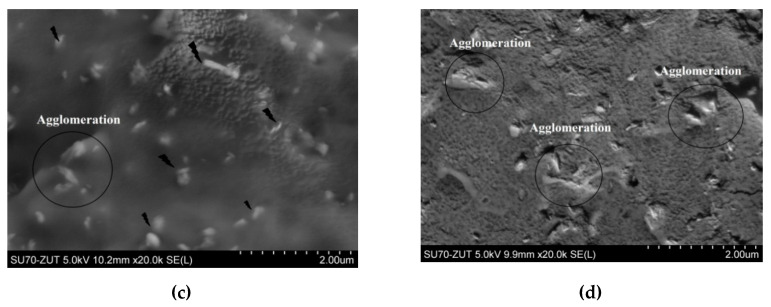
SEM images of PP/HNT NCs (**a**) 1 vol.%, (**b**) 2 vol.%, (**c**) 5 vol.%, and (**d**) 10 vol.% of HNTs.

Figure 3a–d depict the morphology of different content of HNTs (1, 2, 5, and 10 vol. %) in PP matrix. It is clear that at low concentrations of nanofiller (1 and 2 vol.%), HNTs have been regularly dispersed in the polymer matrix and agglomerates were not found. This homogenous distribution confirms that HNTs and PP were well mixed, which might be improved owing to the addition of MAH-g-PP connecting with HNTs. At high concentrations of nanofiller (5 and 10 vol.%), minor agglomeration within the polymer has been observed. The presence of agglomerated clusters leads to a smaller surface area of HNT inclusions per volume, thus weakening the interface strength between HNTs and the PP matrix. These agglomerates act as crack initiators during composite deformation; therefore, their presence corresponds to the reduction in the strength of PP with 10 vol.% of HNTs.

### 3.2. Mechanical Properties

**Figure 4:**
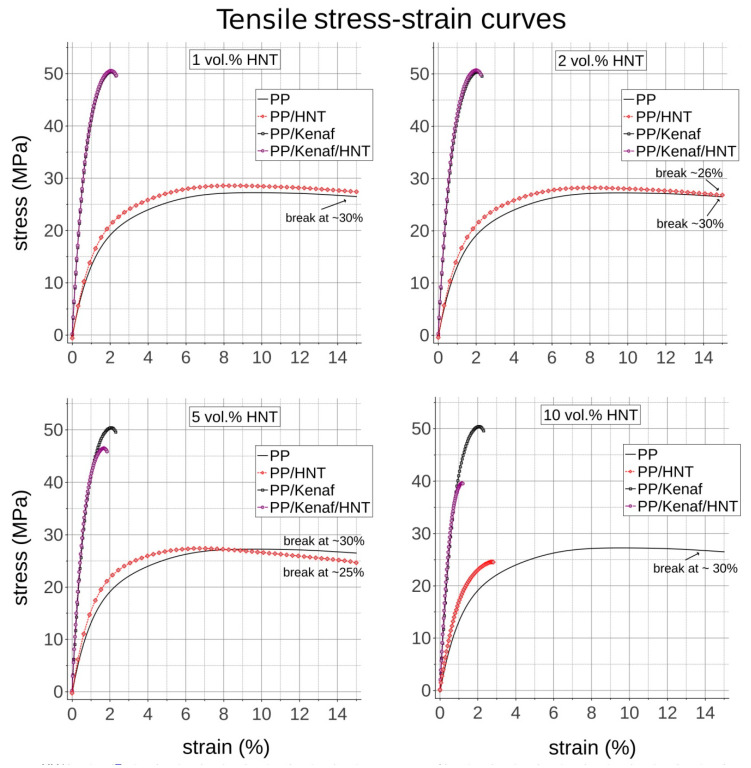
Tensile stress–strain curves for PP, PP/HNT, PP/kenaf, and PP/kenaf/HNT reinforced with different volumetric contents of 1, 2, 5 and 10 vol. % of HNT.

Figure 4 depicts the averaged stress–strain curves of manufactured composites tested in tensile mode. The Young’s modulus of PP increases after the addition of HNTs. Already after adding 1 vol.% of HNTs, the gain of Young’s modulus is 14% concerning the neat PP matrix. The higher the content of HNTs, the greater the Young’s modulus of PP/HNTs NCs, which is increased by ~60% with 10 vol.% of HNTs. The Young’s modulus of PP increases up to ~260% with the addition of 30 vol.% of short kenaf fibers in PP. Nevertheless, in the case of hybrids, the addition of HNTs increases further the Young’s modulus of the composite by 3%, 5%, 9%, and 13% in the case of adding the 1, 2, 5, and 10 vol.% of HNTs, respectively. The increase in Young’s modulus is attributed to Young’s modulus of the filling material, its volumetric content, and shape. It is calculated for very low deformations; therefore, it is not affected by the quality of filler–matrix interphase or the presence of agglomerates. However, these two factors play a more vital role in tensile strength [27]. It can be seen in Table 2 that the tensile strength of PP/HNTs NCs is enhanced by 5% and 3% with the addition of 1 and 2 vol.% HNTs to the pure PP matrix, respectively. Their ductility is also not reduced as they all break at around ~30% of strain. This gain can be attributed to reduced creep by the presence of HNT nanoclay or increased crystallinity owing to the nucleating effect of HNTs [28,29,30]. However, further addition of HNTs diminished the gain and for 10 vol.% content of HNTs, the tensile strength is even reduced by 7%, while the strain to break is reduced to 2.8%. The decrease in ductility of PP with a higher content of HNT nanoclay is related to the low structural ductility of its HNTs and nanoplates. Therefore, even the addition of MAH-g-PP compatibilizer, which develops interphase on the surface of HNTs, does not prevent them from debonding from the PP matrix at higher strains. Furthermore, the higher amount of agglomerates at higher volumetric content of HNTs can be a substantial factor of property loss since agglomerates act as stress risers and debond from the matrix at earlier strains than well-distributed particles with fully developed interphase [27].

Adding 30 vol.% of short kenaf fibers as the main reinforcement increases the tensile strength by 85%. Further addition of HNTs of 1 and 2 vol.% minutely improves this property, but further addition of HNTs to 5 and 10 vol.% reduces tensile strength by 8% and 21%, respectively. This can be an effect of both HNTs’ agglomerates as well as the reduced flow of molten compound during processing (compounding and injection molding) resulting in increased shear forces, which shorten kenaf fibers to a greater extent. It can be therefore concluded that adding higher contents of HNTs is counterproductive and the addition of 1 and 2 vol.% of HNTs into the matrix gives a desirable effect on tensile performance, as shown in Figure 4.

**Figure 5:**
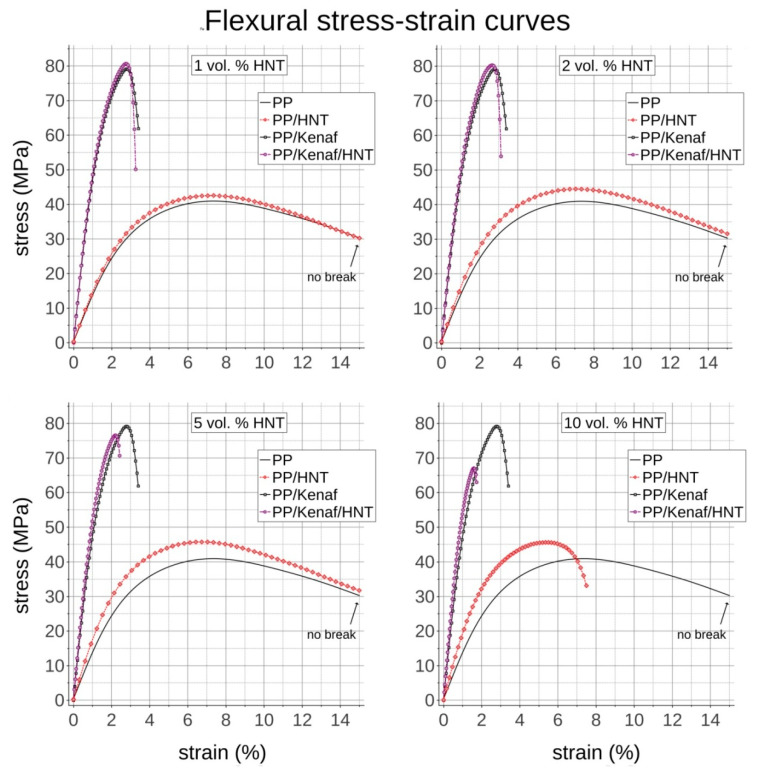
Flexural stress–strain curves for PP, PP/HNT, PP/kenaf, and PP/kenaf/HNT reinforced with different volumetric content of 1, 2, 5 and 10 vol. % of HNT.

Figure 5 presents the averaged flexural stress–strain curves of all manufactured composites evaluated in a 3-point bending test. The flexural modulus rises after the addition of HNTs in a similar manner as in the case of the tensile Young’s modulus. The flexural modulus of NCs with respect to native PP is improved by 7%, 17%, 30%, and 44% for 1, 2, 5, and 10 vol.% of HNTs, respectively. The reference biocomposite reinforced with 30 vol.% of short kenaf fibers gives, however, almost a 3-fold increase in flexural modulus and further addition of HNTs improves it by 3%, 4%, 11%, and 19% for 1, 2, 5, and 10 vol.%, respectively. Flexural strength is increased in PP/HNTs composites by 4%, 9%, and 12% for 1, 2, and 5 vol.% of HNTs, respectively. For 10 vol.% of HNTs, the flexural strength is slightly lower than for 5 vol.%, and the ductility of nanocomposite is reduced as it starts to break at ~7% strain, while other NCs were not breaking at up to 15% of testing strain. However, it is seen that the improvement in flexural strength is higher than in the case of tensile strength, which is an effect of the mode of the 3-point bending test, in which the upper volume of the bent bar is subjected to compression, while the lower part is subjected to straining. This is a typical behavior observed even by filling the material with high-modulus low aspect ratio particles [27]. The twice higher flexural strength than the pure PP matrix is obtained by reinforcing it with 30 vol.% of short kenaf fibers. By the addition of HNT nanoclay to the PP/kenaf biocomposite, the gain in terms of flexural strength is as minor as in the case of tensile strength. Namely, improvement by ~2% is achieved with up to 2 vol.% of HNTs and higher contents reduce the flexural strength and strain to breakage. This, as mentioned before, is likely to be caused by shortening of kenaf fibers induced by reduced melt flow by composite processing at higher filler contents.

### 3.3. Impact Properties

**Figure 6:**
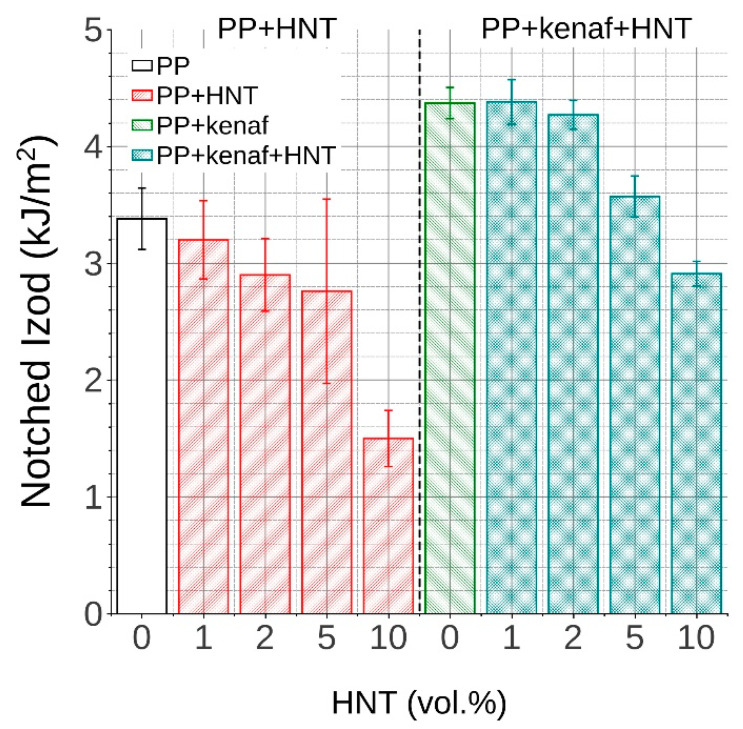
Izod impact strength of PP, PP/HNTs NCs, PP/kenaf, and PP/kenaf/HNTs hybrid NCs reinforced with different volumetric contents of HNTs.

Figure 6 shows a comparison between the Izod notched impact strength of all manufactured NCs, hybrid biocomposites, and the reference biocomposite and pure PP matrix. It can be seen that the addition of HNTs reduces notched impact strength. The reduction is greater the higher the content of HNTs is in polypropylene. It is reduced by 5%, 14%, 18%, and 56% for nanocomposites with 1, 2, 5, and 10 vol.% of HNTs, respectively. Although, in some cases, the nanofillers improve the impact strength by increased crystallinity or a favorable change in crystal morphology [28], as well as other mechanisms reducing microcracking [31,32], their high modulus combined with low aspect ratio may also lead to reduction in impact strength, as in the case of reinforcing with fillers of higher magnitude [26,27]. In turn, reinforcing the PP matrix with short kenaf fibers gives ample rise of notched impact strength by 29%. This is a typical gain, which can be obtained with this amount of kenaf fibers in the PP matrix when a MAH-g-PP compatibilizer is used [33]. The improvement is kept by an additional 1vol.% content of HNTs; however, further addition diminishes the achieved gain by 2%, 18%, and 33% for 2, 5, and 10 vol.% of HNTs, respectively. This can be caused by already mentioned agglomerates of HNTs as well as the increased shortening of kenaf fibers.

### 3.4. Creep Test

The results of creep testing are given in Table 3. The first mode of testing, in which the sample was strained up to 1% of strain and held for 10 min, shows that the HNT-based NCs achieve up to 33% higher stresses than the neat PP matrix. The stresses reached by nano-biocomposites are way higher and are almost thrice higher than for the unreinforced PP matrix. For hybrids, it can be observed that 1 and 2 vol.% addition of HNT increases the stress by 1% and 2%, respectively, which was already observed at tensile testing. However, it is the second mode of testing at which samples were strained until they reached a set value of stresses, which was held for 10 min, that shows a clear reduction in creep under load. The reached strains for PP/HNTs NCs after subjecting them to 20 MPa stress in 10 min timespan are reduced by 12%, 18%, 27%, and 39% for 1, 2, 5, and 10 vol.% of HNT contents, respectively. In hybrids that were subjected to 35 MPa of stress for 10 min, the strain with respect to the reference PP/kenaf biocomposite is reduced by 2%, 6%, 3%, and 13% for 1, 2, 5, and 10 vol.% of HNTs, respectively. It must be however noted that the hybrid with the addition of 10 vol.% of HNT broke before reaching 10 min exposure to 35MPa stress. Therefore, only hybrids with additional 1 and 2 vol.% of nanoclay were tested for the stress of 50 MPa. In that testing, the aforementioned hybrids obtain a reduction in strain by 8% and 13%. This experiment proved that nanoclay reduces creep under the load of the matrix, which was found in previous research work by Merijs-Meri et al., among others [34,35]. The reduction in creep enables the reaching of higher material strengths because it reduces the relaxation of stresses; however, the additional content of nanofiller may lead to shortening of primary reinforcing fibers during processing. This is faced in the hybrid composites with higher contents of nanofiller; therefore, the benefit of reduced creep under load becomes overcome with the negative effect of shortening of kenaf fibers and the occurrence of HNT agglomerates.

### 3.5. Thermal Stability

**Figure 7:**
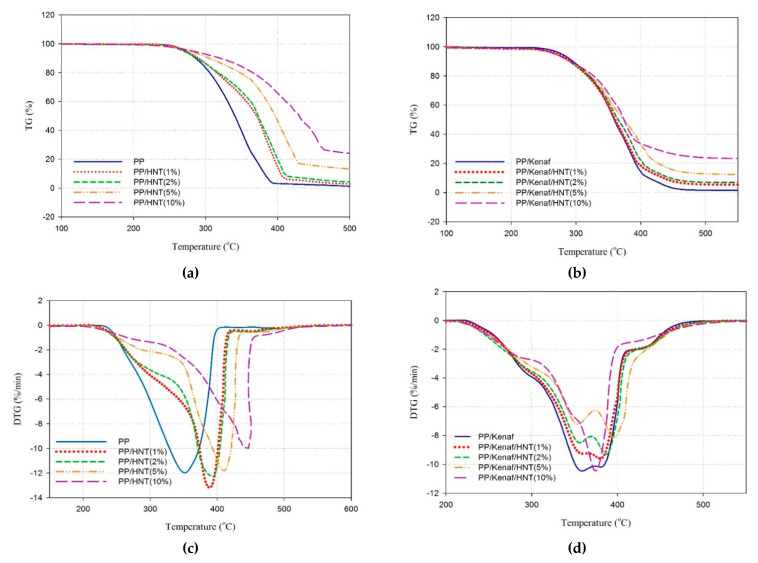
The thermo-oxidative degradation analysis curves for PP and its NCs in air atmosphere (**a**,**b**) weight loss and (**c**,**d**) derivative of weight loss.

Weight loss and its derivative curves for PP, PP/HNTs NCs, and PP/kenaf/HNT nano-biocomposites in an oxidizing atmosphere have been plotted in Figure 7a–d. Moreover, Table 4 presented the temperatures related to the 5%, 10%, and 50% of mass loss for PP, PP/kenaf, and its NCs. Neat PP shows a mass loss of 5% at 271 °C. The thermal stability of PP was improved with the addition of HNTs in the matrix. One can observe that at 10% and 50% of weight loss, the values of temperatures increase along with the increase in the content of HNTs. In Figure 7b, the shift in values of derivative of mass loss for the NCs is because one of the components has higher thermal stability. As represented in Table 4, the thermal stability of the PP/kenaf improved from 359 to 374 °C at 50% of mass loss, along with the addition of 5 vol.% of HNT.

## 4. Conclusions

HNTs used as an additional component to short kenaf fibers in hybrid nano-biocomposites present potential for improvement of their mechanical properties. The reduction in creep by the addition of HNT is the main factor for this improvement. It must be, however, heeded that the addition of higher amounts of HNTs leads to increased shortening of fibers during the processing of such hybrid composites which, in turn, results in the reduction in their mechanical performance. These two opposing effects of nanoclay addition shall be, therefore, taken into account while looking for their optimal content in developed hybrid composites. The manufactured hybrid nano-biocomposites with additional 1 and 2 vol.% of HNTs have slightly improved Young’s modulus and strengths as a result of noticeably reduced creep. Moreover, they maintained the notched impact strength of their reference biocomposite without HNT. Moreover, the combination of HNTs and kenaf fibers caused the improvement of the thermal stability of PP, especially at higher temperatures. It may be assumed that other grades of nanoclays have the potential of a more significant increase in properties and that the proposed route of manufacturing using a nanoclay masterbatch during compounding presents a facile way for developing of these emerging hybrid nano-biocomposites.

## Figures and Tables

**Table 1:** Different compositions of PP/HNTs nanocomposites, PP/kenaf/HNTs nano-biocomposites, and reference PP/kenaf fiber biocomposites.

Specimens	PP (V%)	MAH-g-PP (V%)	Kenaf (V%)	HNT (V%)	PP+MAH-g-PP (wt.%)	Kenaf (wt.%)	HNT (wt.%)
PP	100	0	0	0	100	0	0
PP/HNT(1)	98.92	0.08	0	1	97.26	0	2.74
PP/HNT(2)	97.85	0.15	0	2	94.61	0	5.39
PP/HNT(5)	94.62	0.38	0	5	87.18	0	12.82
PP/HNT(10)	89.25	0.75	0	10	76.31	0	23.69
PP/kenaf(30)	66.75	2.25	30	0	63.78	36.22	0
PP/kenaf(30)/HNT(1)	66.67	2.33	30	1	61.86	35.64	2.5
PP/kenaf(30)/HNT(2)	65.60	2.40	30	2	60.00	35.07	4.93
PP/kenaf(30)/HNT(5)	62.37	2.63	30	5	54.75	33.48	11.77
PP/kenaf(30)/HNT(10)	57	3.00	30	10	46.99	31.13	21.88

**Table 2:** Mechanical properties of PP, PP/HNTs NCs, PP/kenaf, and PP/kenaf/HNTs hybrid biocomposites.

Specimens	Young’s Modulus (GPa)	Tensile Strength (MPa)	Tensile Strain (%)	Flexural Modulus (GPa)	Flexural Strength (MPa)
PP	1.55 ± 0.08	27.2 ± 0.6	29.5 ± 1.5	1.42 ± 0.09	40.9 ± 0.8
PP/HNT(1)	1.76 ± 0.09	28.6 ± 0.4	29.8 ± 2.4	1.52 ± 0.04	42.5 ± 0.8
PP/HNT(2)	1.77 ± 0.04	28.2 ± 0.3	26.0 ± 8.9	1.65 ± 0.05	44.4 ± 0.4
PP/HNT(5)	1.86 ± 0.11	27.3 ± 0.1	25.0 ± 8.3	1.84 ± 0.07	45.7 ± 0.4
PP/HNT(10)	2.46 ± 0.35	25.5 ± 0.3	2.8 ± 0.2	2.05 ± 0.05	45.6 ± 0.8
PP/kenaf(30)	5.70 ± 0.27	50.4 ± 0.3	2.3 ± 0.1	5.46 ± 0.06	79.2 ± 0.8
PP/kenaf(30)/HNT(1)	5.87 ± 0.23	50.6 ± 0.4	2.3 ± 0.1	5.64 ± 0.05	80.9 ± 0.8
PP/kenaf(30)/HNT(2)	6.00 ± 0.05	50.7 ± 0.5	2.2 ± 0.1	5.66 ± 0.10	80.7 ± 0.4
PP/kenaf(30)/HNT(5)	6.23 ± 0.24	46.5 ± 0.6	1.8 ± 0.1	6.08 ± 0.02	76.6 ± 0.4
PP/kenaf(30)/HNT(10)	6.44 ± 0.23	39.6 ± 0.5	1.2 ± 0.1	6.51 ± 0.08	67.3 ± 0.7

**Table 3:** Creep test results for the PP, PP/HNTs NCs, PP/kenaf biocomposite, and PP/kenaf/HNTs nano-biocomposites.

Specimens	Stress (MPa) Strained to 1% and Held for 10 min	Strain at the End (%) Strained to 20 MPa and Held for 10 min	Strain at the End (%) Strained to 35 MPa and Held for 10 min	Strain at the End (%) Strained to 50 MPa and Held for 10 min
PP	11.1 ± 0.3	2.97 ± 0.17	-	-
PP/HNT(1%)	12.4 ± 0.4	2.62 ± 0.08	-	-
PP/HNT(2%)	12.6 ± 0.3	2.44 ± 0.12	-	-
PP/HNT(5%)	13.1 ± 0.7	2.15 ± 0.03	-	-
PP/HNT(10%)	14.7 ± 0.2	1.81 ± 0.04	-	-
PP/kenaf	32.5 ± 0.3	-	0.98 ± 0.02	2.84 ± 0.64
PP/kenaf/HNT(1%)	32.7 ± 0.3	-	0.96 ± 0.03	2.62 ± 0.86
PP/kenaf/HNT(2%)	33.0 ± 0.4	-	0.92 ± 0.07	2.47 ± 0.74
PP/kenaf/HNT(5%)	32.3 ± 0.4	-	0.95 ± 0.02	-
PP/kenaf/HNT(10%)	28.6 ± 0.2	-	0.85 * ± 0.71	-

* Break before reaching 600 s of holding time.

**Table 4:** Characteristic temperatures of the thermo-oxidative decomposition of the NCs.

Specimens	T_5%_ (°C)	T_10%_ (°C)	T_50%_ (°C)
PP	271	286	340
PP/HNT(1%)	272	289	371
PP/HNT(2%)	269	288	373
PP/HNT(5%)	278	305	396
PP/HNT(10%)	281	320	430
PP/kenaf	278	295	359
PP/kenaf/HNT(1%)	271	291	360
PP/kenaf/HNT(2%)	267	289	365
PP/kenaf/HNT(5%)	266	290	374
PP/kenaf/HNT(10%)	268	291	374
